# Monte Carlo Simulation of the Effect of Melanin Concentration on Light–Tissue Interactions in Reflectance Pulse Oximetry

**DOI:** 10.3390/s25020559

**Published:** 2025-01-19

**Authors:** Raghda Al-Halawani, Meha Qassem, Panicos A. Kyriacou

**Affiliations:** Research Centre for Biomedical Engineering, City St George’s, University of London, London, EC1V 0HB, UK; meha.qassem@city.ac.uk (M.Q.); p.kyriacou@city.ac.uk (P.A.K.)

**Keywords:** Monte Carlo simulation, photoplethysmography, skin pigmentation, oxygen saturation, pulse oximetry

## Abstract

Over the past ten years, there has been an increasing demand for reliable consumer wearables as users are inclined to monitor their health and fitness metrics in real-time, especially since the COVID-19 pandemic. Reflectance pulse oximeters in fitness trackers and smartwatches provide convenient, non-invasive SpO_2_ measurements but face challenges in achieving medical-grade accuracy, particularly due to difficulties in capturing physiological signals, which may be affected by skin pigmentation. Hence, this study sets out to investigate the influence of skin pigmentation, particularly in individuals with darker skin, on the accuracy and reliability of SpO_2_ measurement in consumer wearables that utilise reflectance pulse oximeters. A Monte Carlo model is developed to assess the effect on simulated reflectance pulse oximetry measurements across light, moderate, and dark skin types for oxygen saturation levels between 70 and 100%. The results indicate that a one-algorithm-fits-all calibration approach may be insufficient, and root mean square errors (RMSEs) of at least 0.3956%, 0.9132%, and 8.4111% for light, moderate, and dark skin are observed when compared to transmittance calibration algorithms. Further research is required to validate these findings and improve the performance of reflectance pulse oximeters in real-world applications, particularly in the context of consumer wearables.

## 1. Introduction

There has been a remarkable evolution in consumer wearables driven by advancements in sensor technology, including a growing emphasis on personal health monitoring using reflectance pulse oximeters [1,2]. Reflectance pulse oximeters measure blood oxygen saturation (SpO_2_) using light reflected from tissue rather than transmitted through it, making them suitable for body sites like the forehead or chest. This technology employs light-emitting diodes (LEDs) to emit red and infrared light into the skin and a photodetector to capture the light that is backscattered from the tissue. The relative absorption of these wavelengths by oxygenated and deoxygenated haemoglobin allows for SpO_2_ calculation by computing the ratio of ratios values (R). Unlike transmission-based oximeters, reflectance models are less dependent on thin measurement sites like fingers or earlobes, making them versatile for use in diverse clinical settings. However, despite their growing popularity, several limitations exist. One of the primary challenges is the inherent complexity of accurately capturing physiological signals from the skin’s surface. Consumer-grade devices often face technical constraints in achieving the same level of accuracy and reliability as medical-grade equipment relying primarily on transmittance pulse oximeter technology. Factors such as motion artifacts, ambient light interference, and variations in skin properties are inherent in both modes; however, they can be more pronounced in reflectance pulse oximeters, affecting the quality of the photoplethysmography (PPG) signals [3]. As a result, this tends to introduce inaccuracies in oxygen saturation measurement by pulse oximetry, impacting the reliability of the data provided to users, which is a current subject of concern [4].

Moreover, one of the ambiguous challenges regarding pulse oximeters has been in understanding the impact of skin pigmentation on the accuracy of SpO_2_ measurement, especially in individuals with darker skin. This is a limitation that has been explored in both retrospective and prospective studies [5], whereas the majority have focused on investigating SpO_2_ inaccuracies using transmittance pulse oximeters as a common medical and diagnostic practice [6]. Consequently, there is a notable gap in comprehensive studies examining inaccuracies stemming from skin pigmentation in reflectance pulse oximeters, despite the fact that they rely on interactions with the superficial layers of the skin, including various properties such as pigmentation.

Monte Carlo simulation is a powerful computational technique widely used in biomedical optics to model light–tissue interactions. Its significance lies in its ability to account for complex tissue geometries and stochastic light scattering and absorption events. They are also useful for a range of applications, such as predicting the distribution of tissue chromophores [7], deriving the optical properties of tissue layers and constituents [8], and analysing photon behaviour in complex biological structures including analysis of source-detector separation on output reflectance [9]. Other technologies such as optical tomography also use reflected light to create high-resolution cross-sectional images based on light–tissue interactions occurring in deeper tissue structures. While their application is limited in the context of wearable devices where the primary goal is to achieve accurate, real-time measurements of oxygen saturation at the skin’s surface, understanding the principles of light scattering and absorption, which is the core of optical tomography, remains essential for improving the accuracy of reflectance-based systems. By applying MC simulations to study these interactions, this research can provide insights into addressing the challenges posed by melanin concentration in light-based devices. Table 1 provides a summary of the advantages, limitations, and relevance of different computational approaches for studying the effect of melanin on reflectance pulse oximeter algorithms.

Melanin, the primary pigment responsible for skin colour, plays a pivotal role in light absorption within the visible and near-infrared spectrum, which can be simulated in MC models. Studies have shown that melanin concentration in the skin absorbs light differently, influencing the intensity of the detected signal by the oximeter [10]. In highly pigmented skin, increased melanin concentration leads to higher absorption of light, particularly at shorter wavelengths (e.g., red light). This heightened absorption can alter the reflectance signals detected by pulse oximeters, potentially reducing the accuracy of SpO_2_ measurements. As pulse oximeters rely on the ratio of red to infrared light absorption to estimate SpO_2_, variations in melanin levels across different skin tones may introduce biases, necessitating adjustments in device algorithms to account for these differences. Reflectance pulse oximetry is particularly susceptible to the effects of melanin due to the shorter optical path lengths and increased backscattering in superficial layers of the skin. Unlike transmission oximetry, where light travels through the tissue, reflectance measurements are more influenced by the optical properties of the epidermis and dermis, where melanin is concentrated. Consequently, variations in melanin levels can significantly impact the proportion of light reaching the detector, especially at red wavelengths. With very few Monte Carlo simulations modelling light interaction within tissue and incorporating skin pigmentation as a central variable in reflectance pulse oximetry [11,12], the need for more robust modelling is required to accurately characterise the impact of melanin on reflectance signals and inform device calibration. This research has indicated that the AC/DC ratio, which is the ratio of absorbance between the pulsatile and non-pulsatile components of the PPG from the desired anatomical site, can be compromised with increasing melanin concentration, primarily due to differences in light scattering and absorption during systole and diastole. Based on this phenomenon, the calibration algorithms of pulse oximeters may be impacted as they depend on the AC/DC ratio at red and infrared wavelengths. However, modelling skin pigmentation should be enhanced to provide a more comprehensive understanding of its effect on optical parameters related to SpO_2_ calculation by (a) avoiding the stratification of skin pigmentation based on ethnicity, and (b) incorporating a broader range of pigmentation levels into the analysis. Therefore, the novelty of this study lies in examining the influence of melanin concentration on simulated oxygen saturation in reflective geometry using the Fitzpatrick scale. The aim is to obtain a preliminary understanding of the reliability of wearables when worn by individuals of different skin colour, especially those with darker skin pigmentation.

**Table 1:** Comparison of different computational techniques for modelling light–tissue interactions in biological media.

Method	Advantages	Limitations	Relevance to Study
Monte Carlo simulation (MCS)	Highly accurate for modeling complex tissue structures and heterogeneous tissue properties [13]. Capable of accounting for scattering, absorption, and tissue heterogeneity, providing a statistically detailed simulation of light transport and interaction [14].	Can be computationally expensive, requiring significant computational resources and time [15].	Provides high accuracy and reproducible outcomes by running a very high number of photon iterations, making it ideal for studying their stochastic nature in light–tissue interactions as pigmentation changes.
Finite element method (FEM)	Suitable for solving complex systems particularly in laser-based applications [16,17].	Less accurate in modelling light scattering and absorption compared to MCS [18].	FEM can be less suited for pulse oximetry applications to model the complex scattering events in tissues such as skin.
Finite different method (FDM)	Useful for solving the light diffusion equation in simple tissue models and to achieve a balance between accuracy and computational efficiency [19].	Assumes constant tissue properties, limiting its application in heterogeneous tissues like skin [20].	FDM is more suited for simpler models of tissue and may be faster for initial simulations but lacks the precision needed for accurately modelling light absorption and scattering in tissues like skin with varying pigmentation.
Diffusion approximation	Computationally efficient for modelling light transport in scattering media especially in deep tissue [21].	Less accurate for tissues with high scattering properties [22].	Diffusion approximation is better suited for modelling light in deep tissues rather than superficial layers, which is the main focus of this study. Like FEM, it is also not ideal for predicting radiative transport in turbid media such as the human finger.

## 2. Materials and Methods

**Figure 1:**
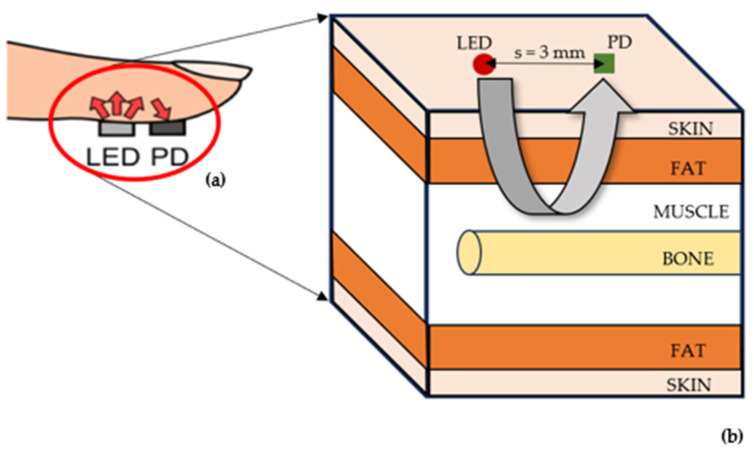
The anatomical site implemented in the developed Monte Carlo model. (**a**) Position of the light-emitting diode (LED) and photodetector (PD) in reflectance mode on the finger. (**b**) The layers of the finger are represented by rectangular slabs (skin, fat, and muscle) and cylinders (bone).

To investigate the effect of melanin concentration on simulated oxygen saturation, a previous Monte Carlo model of the finger was adapted to produce calibration curves for light, moderate, and dark skin in reflectance mode PPG [23]. This involved changing the sensor configuration such that the LED source and photodetector were adjacent to each other, with a separation distance of 3 mm (Figure 1). This source-detector separation was selected as it has shown to produce high-quality AC and DC PPG signals from the finger [24].

Furthermore, the optical properties of all the tissue layers and constituents, including the absorption coefficients ($\mu_{a}$), scattering coefficients ($\mu_{s}$), and anisotropy values (g) at the two operating wavelengths of pulse oximetry (660 nm and 940 nm), as well as the diastolic blood volumes and water concentration values that were inputted into this model can be found from previous work as extracted from the literature [23]. The melanosome fractions ($v_{mel}$) for the three skin types were extracted from the literature to calculate the absorption coefficients of the epidermis, which were 2.55%, 15.5%, and 30.5% for light, moderate, and dark skin, respectively [25]:

(1) $$\mu_{a\_epi}\;\left[{mm}^{-1}\right]=\mu_{a_{\_melanin}}\times v_{mel}+\mu_{a_{w}}\times v_{w}+\left(1-v_{w}-v_{mel}\right)+\mu_{a_{\_skinbaseline}}$$

where

$$\mu_{a_{\_melanin}}=6.6\times 10^{10}\times \lambda^{-3.33}$$

$$\mu_{a_{\_skinbaseline}}=7.84\times 10^{7}\times \lambda^{-3.255}$$

$\mu_{a\_w}$ is the absorption coefficient of water $\left({mm}^{-1}\right)$; and $v_{w}$ is the volume fraction of water.

Furthermore, the systolic phase was replicated by doubling the diastolic blood volume, which was equally distributed between arterial and venous blood [26]. Changes in arterial and venous blood oxygenation were accounted for when calculating the absorption coefficients of arterial and venous blood using the following equation:

(2) $$\mu_{a_{derm\left(n\right)}}\;\left[{mm}^{-1}\right]={sat}_{A}\times \mu_{a_{Hbo}}+\left(1-{sat}_{A}\right)\times \mu_{a_{HHb}}\times v_{A}+{sat}_{V}\times \mu_{a_{Hbo}}+\left(1-{sat}_{V}\right)\times \mu_{a_{HHb}}\times v_{V}+\;\mu_{a_{w}}\times v_{w}+(1-\left(v_{A}+v_{V}+v_{w}\right))\times 7.84\times 10^{7}\times \lambda^{-3.255}$$

where

${sat}_{A}$ is arterial oxygen saturation, ranging between 70% and 100% in increments of 5%;

${sat}_{V}$ is venous oxygen saturation, set to 10% less than ${sat}_{A}$;

$\left(1-{sat}_{A}\right)$ and $(1-{sat}_{v})\times \mu_{a_{HHb}}$ is the contribution from deoxygenated arterial and venous blood, respectively;

derm(n) is the nth sublayer of the dermis;

λ is the wavelength of interest (nm);

$v_{v}$ is the volume fraction of venous blood;

$v_{A}$ is the volume fraction of arterial blood;

$\mu_{a_{Hbo}}$ is the absorption coefficient of oxyhaemoglobin $\left({mm}^{-1}\right)$;

and $\mu_{a_{HHb}}$ is the absorption coefficient of deoxyhaemoglobin $\left({mm}^{-1}\right)$.

The model was run until one million photons were detected. Output reflectance, given by the ratio of incident and output intensity, was calculated during systole and diastole to compute the AC and DC components at both wavelengths. The perfusion index (PI) was calculated by dividing the AC value by the DC value for both wavelengths. This allowed for calculation of the ratio of ratios at each oxygen saturation level, by dividing the perfusion index at 660 nm by the perfusion index at 940 nm.

## 3. Results and Discussion

**Figure 2:**
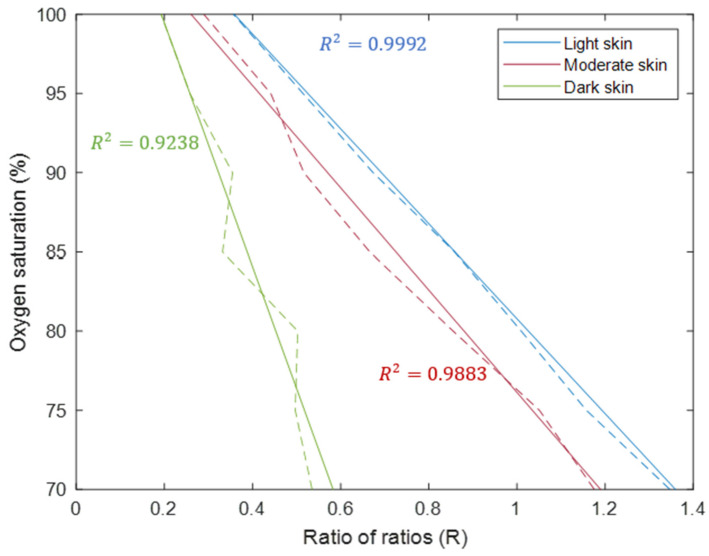
Simulated calibration curves for light, moderate, and dark skin. The dashed lines show the calculated ratio of ratios values from the raw intensity data, and the solid lines show the lines of best fit.

The Monte Carlo model presents three distinct calibration curves for light, moderate, and dark skin (Figure 2). These curves are derived by computing the ratio of ratios using input and output intensity data during systole and diastole. From this data, it is clear that red light is highly absorbed by melanin in comparison to infrared light, especially at higher melanin concentrations. As a result, normalised reflectance at 660 nm reduces in magnitude as melanin concentration increases, and more so at lower oxygen saturation levels. With larger and less compromised normalised reflectance values at 940 nm across the simulated oxygen saturation range, a consistent reduction in the range of the ratio of ratios is observed. Moderate and dark skin calibration curves are shifted with respect to the light skin calibration curve due to changes in skin pigmentation for the same oxygen saturation level, thereby leading to inaccuracies and especially overestimation of SpO_2_.

**Figure 3:**
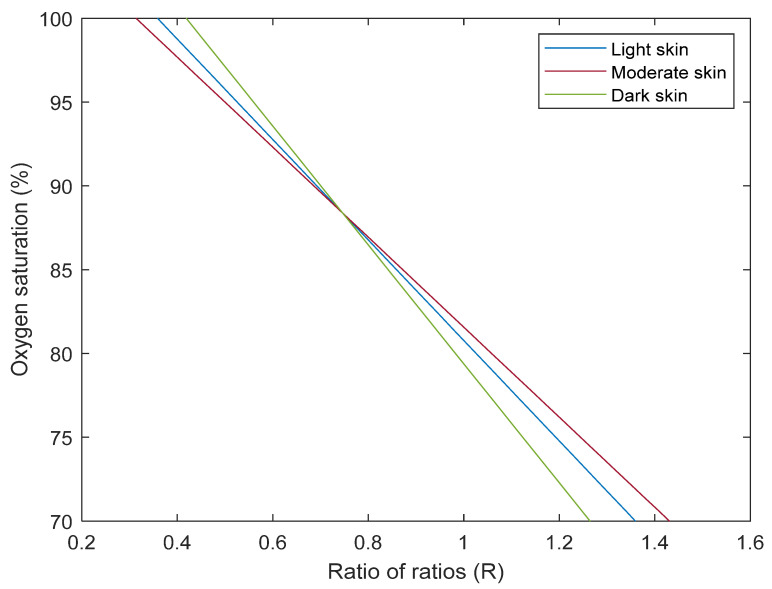
Adjustment of moderate and dark skin simulated calibration curves relative to light skin with applied corrective factors.

Once again, the results indicate that a single calibration algorithm may not allow for the accurate measurement of health metrics such as SpO_2_ by smart wearable technologies on diverse skin pigmentations as is the case with transmittance pulse oximeters [23]. Therefore, a similar approach of applying correction factors to the moderate and dark skin calibration curves was adopted. In doing so, correction factors of 1.21 and 2.17 were calculated, respectively, and applied to the original calibration curves (Figure 3). As shown, the simulated curves do not fully overlap with each other across the entire oxygen saturation range, unlike the behaviour observed with previous work on transmittance pulse oximeters. This discrepancy is possibly due to differences in light absorption and scattering that arise from variations in skin pigmentation as photon travel is primarily limited to the superficial layers, and where sensitivity could be heightened in the epidermis region from backscattering [27]. Therefore, in reflectance mode, there is a greater likelihood for photons to traverse a shorter depth before they are detected especially in darker skin types due to the increased absorption by higher melanin levels, and more so at shorter wavelengths. Hence, additional and/or alternative corrections could be explored by adjusting the source-detector separation for different skin types. By varying the separation distances for individuals with light, moderate, and dark skin, similar photon penetration depths can be achieved, which could ensure more consistent light interaction with specific regions of the tissue. This adjustment could help to normalise the calibration curves, potentially making post-processing corrective measures easier to implement.

**Figure 4:**
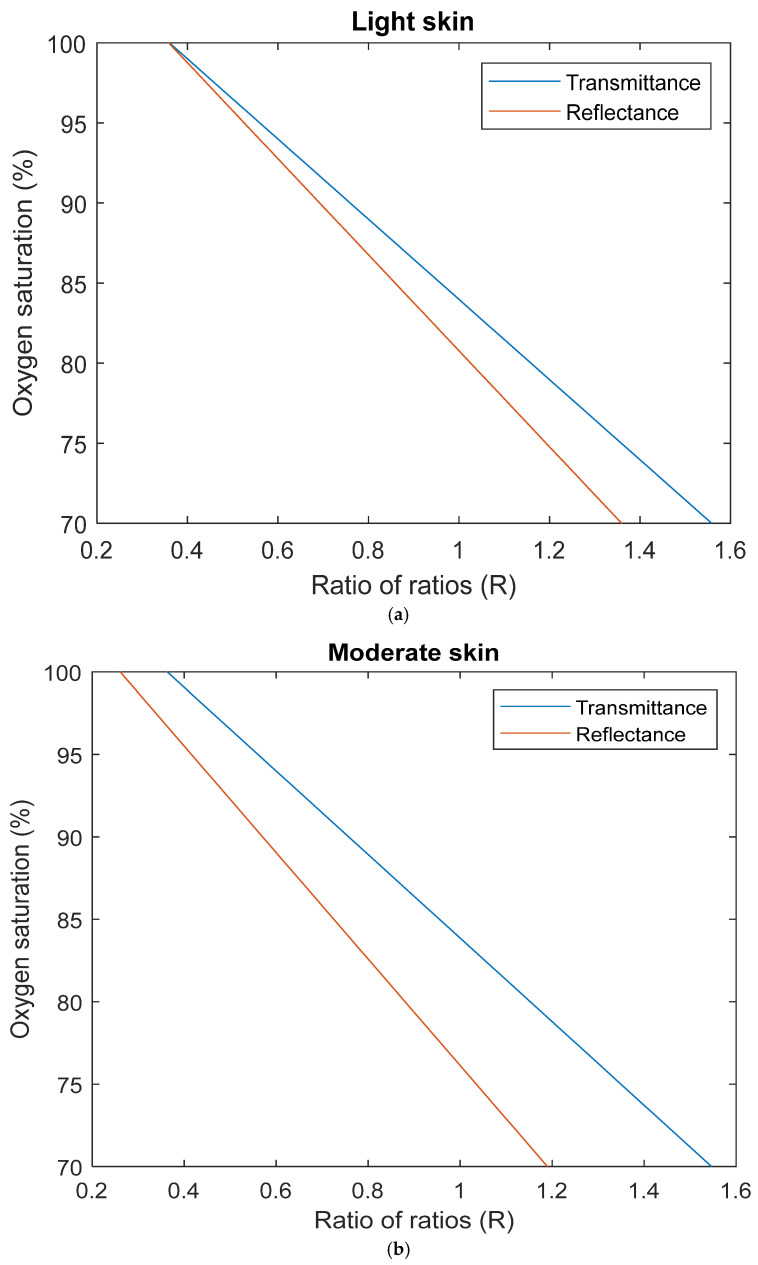

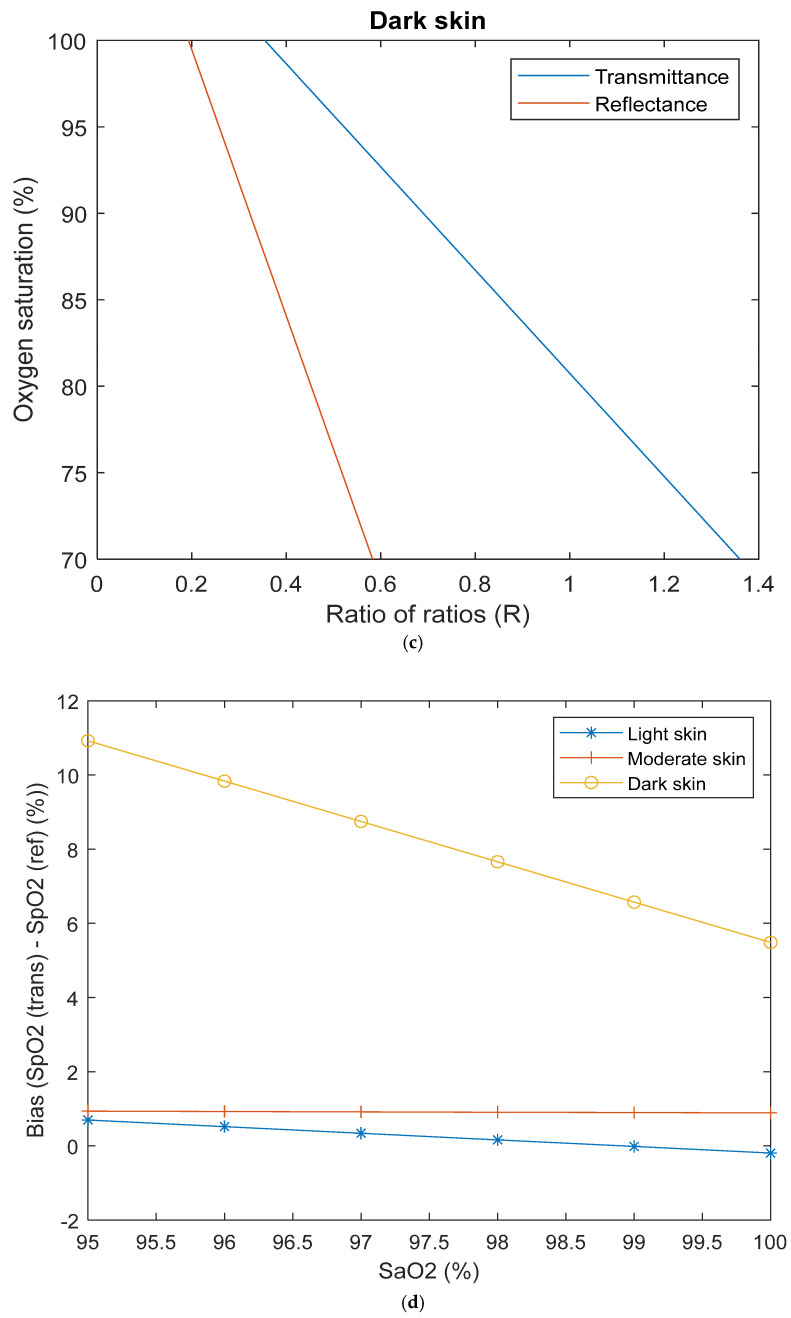
Simulated calibration curves in transmittance and reflectance mode pulse oximetry for (**a**) light skin, (**b**) moderate skin, and (**c**) dark skin. (**d**) Calculated bias between simulated transmittance and reflectance SpO_2_ for healthy SaO_2_ range (95–100%).

From the existing literature, it is known that reflectance pulse oximeters exhibit a decreased accuracy level of 3.5% compared to transmittance pulse oximeters of 3% [28]. Hence, part of the data analysis was to assess whether the computational outcomes reflect this accuracy gap by comparing simulated calibration algorithms for light, moderate, and dark skin in transmittance mode [23] (Figure 4). Visually, the simulated calibration curves suggest that the effect of melanin concentration on oxygen saturation measurement was significantly amplified in reflectance mode, and that pulse oximeter calibrations may indeed be calibrated on individuals from predominantly light-skinned populations due to the smaller gap between both modes. Furthermore, this accuracy consistently decreased with oxygen saturation as observed in studies on hypoxaemic patients [29,30]. All simulated calibration curves were defined by an equation with a linear function, provided in Table 2.

Next, these equations were used to calculate the level of bias between reflectance and transmittance modes for each skin type i.e., light skin transmittance—light skin reflectance, etc. To do this, the equation derived from the simulated light skin transmittance pulse oximetry curve was used as a reference as it closely matched the commonly used commercial pulse oximeter algorithm. This equation was rearranged to calculate the ratio of ratios values for a healthy SaO_2_ range of 95–100% to understand the effect of melanin concentration independently of SpO_2_ inaccuracies at lower oxygen saturation levels. These R values were then substituted into all other equations to calculate bias as shown in Figure 4d. Evidently, light and moderate skin calibration exhibit a low level of bias across the SaO_2_ range between −0.0154% and 0.6984%, and between 0.8884% and 0.9376%, respectively. This results in a root mean square error (RMSE) of 0.3956% and 0.9132% in comparison to 8.4111% for dark skin. However, it is important not to interpret this error by its actual value until further computational investigation of dark skin simulation is conducted due to its more complex structure. Rather, this indicates the potential difference in bias between transmittance and reflectance pulse oximeters on skin types with high melanin concentrations.

Following development of the model and conducting simulations to explore light–tissue interactions of a perfused finger with three distinct skin types, it is clear that a single calibration curve for measuring oxygen saturation is inadequate without accounting for variations in skin pigmentation. This study underscores the need to rectify inaccuracies inherent in reflectance pulse oximeters, particularly due to the high demand of consumer wearables.

In light of these findings, other computational studies have adopted a similar approach in attempt to provide insight into the variations in SpO_2_ measurements based on differences in skin pigmentation. For instance, Bolic conducted an examination of the influence of skin colour and the depth of the air gap on SpO_2_ levels in reflectance mode, focusing on melanin concentrations ranging from 0.3% to 16% [11]. Similar trends in the simulated calibration algorithms were observed, specifically higher levels of absorption at 660 nm as melanin concentration increased. Some limitations of the study included an anomalous calibration outcome at 8% melanin concentration, whereby a satisfactory explanation was not provided. Additionally, the study focused on the exclusive representation of light and moderate skin types, which neglects individuals with darker skin tones [25], including a significant portion of African populations. Therefore, this could explain the smaller difference in the y-intercepts of the best fit lines, similar to the differences between light and moderate skin in the current study with 2.55% and 15.5% melanin concentrations, respectively.

Furthermore, Jung et al. [12] conducted a study to characterise and evaluate the estimation of oxygen saturation measured by a wrist-worn reflectance pulse oximeter during sleep. While the study had a number of limitations, the accuracy of the smartwatch was said to comply with FDA and ISO standards. In the context of skin pigmentation as the limitation explored in this study, the 97 participants enrolled in the investigation were reported as ‘Asian’. While ethnicity may not serve as a precise method for stratifying skin pigmentation, the probability of not meeting FDA and ISO standards was low, assuming uniform skin pigmentation levels across all participants and a maximum RMSE of ±8% with co-oximetry as a reference. Therefore, future studies are advised to recruit an adequate number of participants with quantified skin pigmentation levels and assess SpO_2_ measurement using reflectance pulse oximeters. This can, in addition to providing insight into the effect of skin pigmentation in reflectance pulse oximeters, serve as a validation study to compare with the simulated results from the current Monte Carlo model. While this has been investigated by Jiang et al. [19], larger sample sizes and raw SpO_2_ data are required for a more direct comparison. Meanwhile, the generated data from the MC model should be interpreted with caution as there is still potential to enhance the modelling of haemodynamics and skin colour more objectively.

## 4. Conclusions

This study investigated the impact of skin pigmentation on the accuracy of reflectance pulse oximetry using a Monte Carlo model. By simulating oxygen saturation measurements across light, moderate, and dark skin types, the results highlight significant challenges in applying a single calibration algorithm for all skin tones. Increased melanin concentrations were shown to attenuate red light reflectance disproportionately, leading to biases in SpO_2_ estimations, particularly for individuals with darker skin. Root mean square errors were markedly higher for dark skin compared to light and moderate skin when using transmittance-based calibration algorithms as a benchmark. These findings underscore the limitations of current reflectance pulse oximeters and the necessity of tailored calibration algorithms to ensure accuracy and inclusivity in consumer wearables. Future work should validate these findings through experimental studies with diverse populations and optimise device algorithms to address disparities in SpO_2_ measurement caused by variations in skin pigmentation.

## Figures and Tables

**Table 2:** Calibration equations for the Monte Carlo simulated curves from finger transmittance and reflectance pulse oximetry with light, moderate, and dark skin. The ratio of ratios is given by ‘*R*’.

	Transmittance	Reflectance
Light skin	SaO_2_ = 109 − 25.44 × R	SaO_2_ = 110.8 − 29.98 × R
Moderate skin	SaO_2_ = 109.2 − 32.05 × R	SaO_2_ = 108.4 − 32.3 × R
Dark skin	SaO_2_ = 110.6 − 49.33 × R	SaO_2_ = 114.9 − 76.99 × R
Commercial	SpO_2_ = 110 − 25 × R	

## Data Availability

The code and some of the data that support the findings of this article are not publicly available due to privacy. Other data not included in this article can be requested from the author at raghda.al-halawani@city.ac.uk.

## References

[1] Kang H.S., Exworthy M. (2022). Wearing the Future—Wearables to Empower Users to Take Greater Responsibility for Their Health and Care: Scoping Review. JMIR mHealth uHealth.

[2] Ferreira J.J., Fernandes C.I., Rammal H.G., Veiga P.M. (2021). Wearable technology and consumer interaction: A systematic review and research agenda. Comput. Hum. Behav..

[3] Scardulla F., Cosoli G., Spinsante S., Poli A., Iadarola G., Pernice R., Busacca A., Pasta S., Scalise L., D’Acquisto L. (2023). Photoplethysmograhic sensors, potential and limitations: Is it time for regulation? A comprehensive review. Measurement.

[4] Hosseini M.M., Hosseini S.T.M., Qayumi K., Hosseinzadeh S., Tabar S.S.S. (2023). Smartwatches in healthcare medicine: Assistance and monitoring; a scoping review. BMC Med. Inform. Decis. Mak..

[5] Singh S., Bennett M.R., Chen C., Shin S., Ghanbari H., Nelson B.W. (2024). Impact of Skin Pigmentation on Pulse Oximetry Blood Oxygenation and Wearable Pulse Rate Accuracy: Systematic Review and Meta-Analysis. J. Med. Internet Res..

[6] Al-Halawani R., Charlton P.H., Qassem M., Kyriacou P.A. (2023). A review of the effect of skin pigmentation on pulse oximeter accuracy. Physiol. Meas..

[7] Chen R., Huang Z., Lui H., Hamzavi I., McLean D.I., Xie S., Zeng H. (2007). Monte Carlo simulation of cutaneous reflectance and fluorescence measurements—The effect of melanin contents and localization. J. Photochem. Photobiol. B Biol..

[8] Cook P.D., Bixler J.N., Thomas R.J., Early E.A. (2020). Prediction of tissue optical properties using the Monte Carlo modeling of photon transport in turbid media and integrating spheres. OSA Contin..

[9] Chatterjee S., Kyriacou P.A. (2019). Monte Carlo Analysis of Optical Interactions in Reflectance and Transmittance Finger Photoplethysmography. Sensors.

[10] Keller M.D., Harrison-Smith B., Patil C., Arefin M.S. (2022). Skin colour affects the accuracy of medical oxygen sensors. Nature.

[11] Bolic M. (2023). Simulating the Effects of Melanin and Air Gap Depth on the Accuracy of Reflectance Pulse Oximeters. Proceedings of the 16th International Joint Conference on Biomedical Engineering Systems and Technologies.

[12] Jung H., Kim D., Lee W., Seo H., Seo J., Choi J., Joo E.Y. (2022). Performance evaluation of a wrist-worn reflectance pulse oximeter during sleep. Sleep Health.

[13] Zhu C., Liu Q. (2013). Review of Monte Carlo modeling of light transport in tissues. J. Biomed. Opt..

[14] Krasnikov I., Seteikin A., Roth B. (2019). Advances in the simulation of light–tissue interactions in biomedical engineering. Biomed. Eng. Lett..

[15] Nikolaidis E., Pandey V., Mourelatos Z. (2012). Managing the Computational Cost in a Monte Carlo Simulation by Considering the Value of Information. Proceedings of the SAE 2012 World Congress & Exhibition.

[16] Klanecek Z., Hren R., Simončič U., Muc B.T., Lukač M., Milanič M. (2023). Finite Element Method (FEM) Modeling of Laser-Tissue Interaction during Hair Removal. Appl. Sci..

[17] Kim H.-J., Um S.-H., Kang Y.G., Shin M., Jeon H., Kim B.-M., Lee D., Yoon K. (2023). Laser–tissue interaction simulation considering skin-specific data to predict photothermal damage lesions during laser irradiation. J. Comput. Des. Eng..

[18] Jiang J., Ren W., Isler H., Kalyanov A., Lindner S., Aldo D.C.M., Rudin M., Wolf M., Ryu P.-D., LaManna J.C., Harrison D.K., Lee S.-S. (2020). Validation and Comparison of Monte Carlo and Finite Element Method in Forward Modeling for Near Infrared Optical Tomography. Oxygen Transport to Tissue XLI.

[19] Ortega-Quijano N., Romanov O.G., Fanjul-Vélez F., Salas-García I., Tolstik A.L., Arce-Diego J.L. (2011). Numerical modeling of light propagation in biological tissues: Time-resolved 3D simulations based on light diffusion model and FDTD solution of Maxwell’s equations. Proceedings of the European Conference on Biomedical Optics.

[20] Hielscher A.H., Alcouffe R.E., Barbour R.L. (1998). Comparison of finite-difference transport and diffusion calculations for photon migration in homogeneous and heterogeneous tissues. Phys. Med. Biol..

[21] Zhao Y., Raghuram A., Kim H.K., Hielscher A.H., Robinson J.T., Veeraraghavan A. (2021). High Resolution, Deep Imaging Using Confocal Time-of-flight Diffuse Optical Tomography. IEEE Trans. Pattern Anal. Mach. Intell..

[22] Venugopalan V., You J.S., Tromberg B.J. (1998). Radiative transport in the diffusion approximation: An extension for highly absorbing media and small source-detector separations. Phys. Rev. E.

[23] Al-Halawani R., Qassem M., Kyriacou P.A. (2024). Monte Carlo simulation of the effect of melanin concentration on light–tissue interactions for transmittance pulse oximetry measurement. J. Biomed. Opt..

[24] Hickey M., Kyriacou P.A. (2007). Optimal spacing between transmitting and receiving optical fibres in reflectance pulse oximetry. J. Phys. Conf. Ser..

[25] Kanellis V.G. (2019). A review of melanin sensor devices. Biophys. Rev..

[26] Schmitt J.M., Wall R.T., Zhou G.X., Walker E.C. (1990). Multilayer model of photon diffusion in skin. J. Opt. Soc. Am. A.

[27] Moradi M., Vasudevan S., Bhusal A., Weininger S., Chen Y., Pfefer J. (2024). Modeling light-tissue interactions in pulse oximetry: Effect of device design and skin pigmentation. Proceedings of the Design and Quality for Biomedical Technologies XVII.

[28] Rafl J., Bachman T.E., Rafl-Huttova V., Walzel S., Rozanek M. (2022). Commercial smartwatch with pulse oximeter detects short-time hypoxemia as well as standard medical-grade device: Validation study. Digit. Health.

[29] Cabanas A.M., Martín-Escudero P., Shelley K.H. (2023). Improving pulse oximetry accuracy in dark-skinned patients: Technical aspects and current regulations. Br. J. Anaesth..

[30] Feiner J.R., Severinghaus J.W., Bickler P.E. (2007). Dark skin decreases the accuracy of pulse oximeters at low oxygen saturation: The effects of oximeter probe type and gender. Anesth. Analg..

